# A tail of 2 proteins: the cytoplasmic tail of Cf receptor-like proteins modulates effector-triggered activation of cell death

**DOI:** 10.1093/plphys/kiag632

**Published:** 2026-10-13

**Authors:** Josephine H R Maidment

**Affiliations:** Assistant Features Editor, Plant Physiology, American Society of Plant Biologists, United States; PHIM Plant Health Institute, INRAE, CIRAD, Institut Agro, IRD, Université de Montpellier, Montpellier 34980, France; Centre de Biologie Structurale, INSERM, CNRS, Université de Montpellier, Montpellier 34090, France

Plant immune receptor proteins detect molecular signatures of pathogen infection to activate defense responses. Most receptor proteins belong to one of two groups: receptors at the cell surface survey the apoplast, while nucleotide-binding leucine-rich repeat (NLR) receptors detect molecules which have been secreted by a pathogen and translocated into the host cell. Cell surface receptors share an extracellular, ligand-binding, “sensor” domain and a transmembrane domain. They can be further subdivided into receptor-like kinases (RLKs or RKs), which contain an intracellular kinase domain, and receptor-like proteins (RLPs or RPs), which lack a kinase domain and instead typically have a short intracellular C-terminal tail (Fig. 1a).

**Figure 1:**
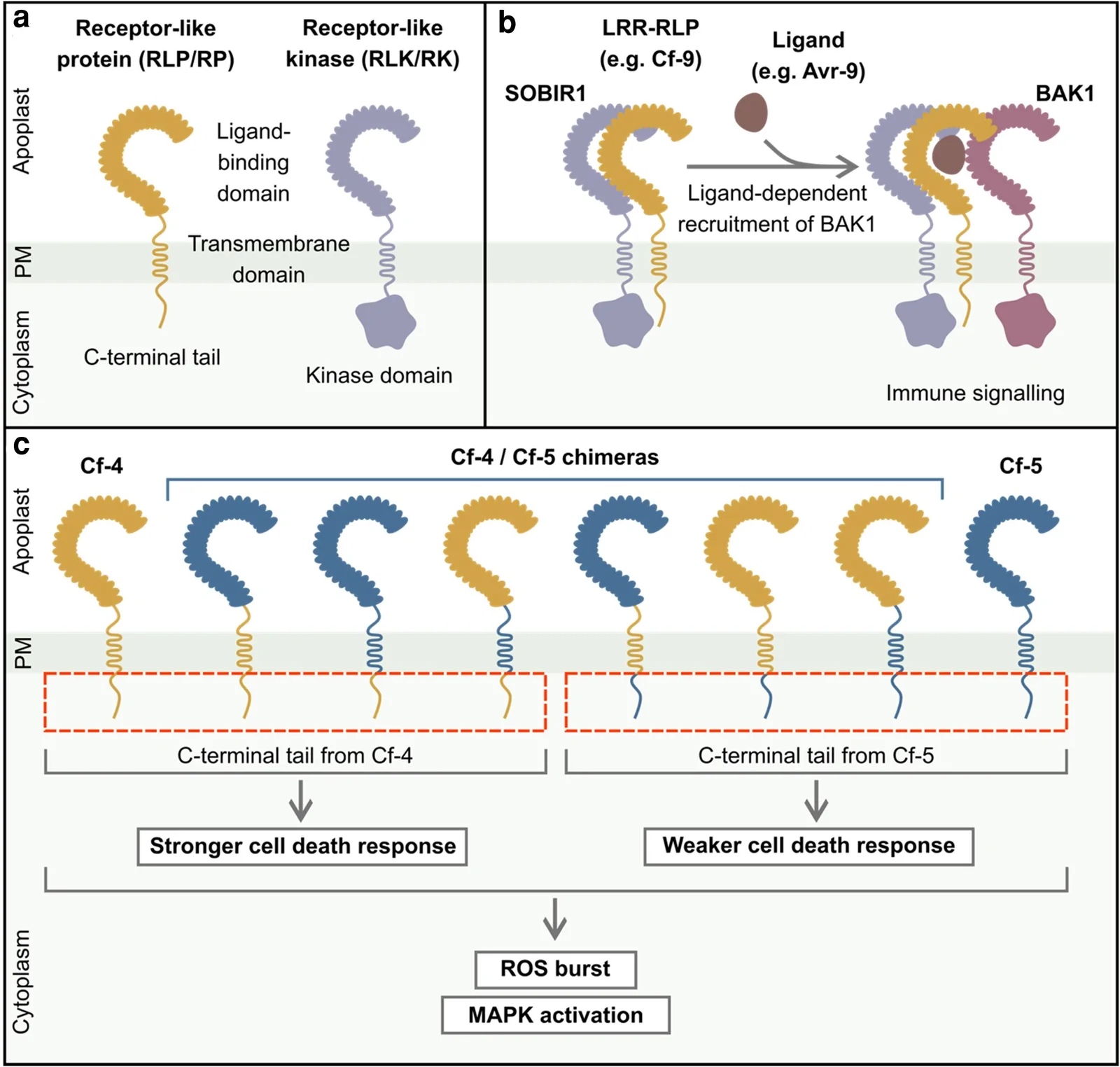
(a) Schematic representation of the domain architecture of receptor-like proteins (RLPs/RPs) and receptor-like kinases (RLKs/RKs). (b) LRR-RLPs, such as Cf-9, constitutively associate with the receptor-like kinase SOBIR1. Following ligand binding, a second receptor-like kinase, BAK1, is recruited, leading to activation of immune signalling. (c) Cf-4, and Cf-4/Cf-5 chimeras carrying the C-terminal tail of Cf-4, activate a stronger cell death response than Cf-5 or Cf-4/Cf-5 chimeras containing the C-terminal tail of Cf-5, yet all chimeras exhibit comparable ROS burst and MAPK activation.

The leucine-rich repeat (LRR) class of RLPs (LRR-RLPs) contains an extracellular LRR domain that typically recognizes proteinaceous ligands. LRR-RLPs constitutively associate with an LRR-RLK, SUPPRESSOR OF BIR1 (SOBIR1) (Liebrand et al. 2013). Following ligand binding to the LRR domain, a second LRR-RLK, BRI1-ASSOCIATED RECEPTOR KINASE 1 (BAK1), is recruited to the complex (Albert et al. 2015) (Fig. 1b). Transphosphorylation events between the 2 kinase domains initiate phosphorylation cascades and activate immune responses such as Ca^2+^ influx, callose deposition, reactive oxygen species (ROS) formation, and transcriptional reprogramming. Some LRR-RLPs engage intracellular NLR receptors to initiate cell death (reviewed by [Kourelis 2023]), which restricts the spread of a pathogen through the plant, while other LRR-RLPs trigger resistance in the absence of cell death. Multiple studies have investigated how different domains of RLPs bind their ligand, engage SOBIR1/BAK1, and activate downstream signalling and immunity. Notably, deletion of the C-terminal tail has been shown to compromise the cell death–inducing activity of RLPs such as RXEG1 (Sun et al. 2022) and AtRLP23 (Albert et al. 2019).

In a recent study published in *Plant Physiology*, Budak et al. (2026) focus on the tomato (*Solanum lycopersicum)* LRR-RLPs Cf-4, Cf-9, Cf-2, and Cf-5. Each of these receptors detects an effector protein, designated Avirulence (Avr) 4, Avr9, Avr2, and Avr5, from the apoplastic fungal pathogen *Fulvia fulva*. Cf-4 and Cf-9 were previously shown to rapidly and robustly trigger cell death in the presence of Avr4 and Avr9, respectively (Cai et al. 2001). In contrast, Cf-2 and Cf-5 were reported to activate chlorosis, rather than cell death, several days after exposure to their cognate fungal effector (Brading et al. 2000). Budak et al. first verified these reported phenotypes in both homologous and heterologous systems (Budak et al. 2026). Tomato plants expressing one of the receptors were infiltrated with apoplastic fluid from a plant infected with *F. fulva* expressing the cognate effector. The receptor/effector combinations Cf-4/Avr4 and Cf-9/Avr9 resulted in a stronger cell death response than Cf-2/Avr2 and Cf-5/Avr5. The same pattern was observed following transient expression of Cf/Avr pairs in the model plant *Nicotiana benthamiana*.

To dissect the contribution of the C-terminal tail and transmembrane domain to the observed cell death response, Budak et al. generated a series of chimeric Cf-4/Cf-5 receptors (Fig. 1c). Each chimera was transiently expressed in *N. benthamiana* alongside the effector corresponding to the LRR domain present in the chimera. Regardless of the extracellular or transmembrane domain, chimeric receptors containing the C-terminal tail of Cf-4 activated strong cell death, while chimeras containing the Cf-5 C-terminal tail triggered a significantly weaker response (Budak et al. 2026) (Fig. 1c). The same pattern was observed for chimeric Cf-4/Cf-2 receptors. Next, they assessed the activation of 2 early immune signalling events, a ROS burst and phosphorylation (and activation) of mitogen-activated protein kinases (MAPKs), following transient expression of Cf-4, Cf-5, and Cf-4/Cf-5 chimeras and their corresponding effector in *N. benthamiana*. No significant difference in ROS burst was observed between Cf-4 and the chimeric Cf-4 proteins, nor between Cf-5 and the chimeric Cf-5 proteins. In addition, no difference in MAPK phosphorylation (as detected with an anti-p42/p44-ERK antibody) was detected between Cf-4/Cf-5 and their respective chimeras (Fig. 1c). While the chimeras were detected at similar levels to the wild-type receptors following immunoprecipitation, it was not possible to detect the proteins by Western blot without enrichment by immunoprecipitation. Therefore, it could not be confirmed that the differences in cell death output were not caused by variation in the accumulation of the receptor proteins. However, the similar ROS burst and MAPK activation triggered by each of the chimeras is consistent with the author's conclusion that the proteins accumulated to similar levels. Together, these data support the model that the C-terminal tail of Cf proteins determines the intensity of the cell death output.

The authors attempted to delimit a region of the C-terminal tail of Cf-4 required for robust activation of cell death. Structural modelling predicted that the C-terminal tail consists of an α-helix flanked by unstructured regions. Deletion of either the α-helix or C-terminal unstructured region significantly reduced the intensity of cell death observed following transient expression of the receptor with Avr4 in *N. benthamiana*. Interestingly, both deletions also attenuated the ROS burst, indicating that while the C-terminal tail of Cf-4 can be exchanged for that of Cf-5 without compromising activation of the ROS burst, an intact C-terminal tail appears necessary for this response. However, it cannot be excluded that these deletions destabilized the proteins and/or reduced their accumulation compared to the full-length receptors.

Finally, Budak et al. investigated whether the differences in cell death triggered by Cf-4/Cf-9 and Cf-2/Cf-5 could be related to differences in association with the adaptor protein SOBIR1. Following transient expression of the Cf proteins with *S. lycopersicum* SOBIR1 in *N. benthamiana* and immunoprecipitation of similar amounts of each Cf receptor, immunoblotting revealed that more SlSOBIR1 was associated with Cf-4/Cf-9 than with Cf-2/Cf-5 (Budak et al. 2026). The same experiment with the Cf-4/Cf-5 chimeras revealed that SlSOBIR1 associated most with Cf-4 and with the Cf-5 chimera containing the transmembrane and Ct regions of Cf-4. In other RLPs, SOBIR1 binding has been shown to depend on a GxxxG motif in the transmembrane domain and/or a negatively charged patch in the extracellular juxtamembrane (eJM) region (Albert et al. 2019). However, the observation that less SOBIR1 protein immunoprecipitated with a Cf-4 chimera where the C-terminal tail alone was replaced with that of Cf-5 suggests that, at least in the case of Cf-4, the C-terminal tail may also contribute to SOBIR1 binding.

Overall, this study contributes to a growing body of work assessing how the different domains of RLPs contribute to immune outputs and disease resistance. Cell surface receptors often recognize conserved molecules found in multiple pathogens, and the transfer of RLKs and RLPs between species has been shown to confer broad-spectrum resistance to different diseases. However, interspecies transfer of cell surface receptors can be complicated by their requirement for compatible co-receptors and downstream signalling components. A recent study highlighted how rational engineering of *Arabidopsis thaliana* RLP23 and subsequent transfer to tomato resulted in robust resistance to multiple pathogens (Yang et al. 2025). Cf-4 requires the intracellular NLR receptor NRC3 to trigger cell death (Kourelis et al. 2022), and the Avr4-dependent cell death induced by Cf-4/Cf-5 chimeras was also NRC2/3 dependent (Budak et al. 2026). It remains unclear whether Cf-4 physically interacts with NRC3, but together these studies suggest that the C-terminal tail of Cf-4 conditions NRC3 activation. Understanding the molecular detail of how RLPs activate specific immune responses will provide a foundation for future work to engineer these receptors to deliver robust resistance to plant pathogens.

## Recent related articles in *Plant Physiology*

- (Hailemariam et al. 2026) revealed the role of a tomato receptor-like cytoplasmic kinase in integrating plant responses to pathogens and to light stress.
- (Chen et al. 2026) described a pathogen-inducible peptide in rice that activates immune responses in a range of plant species.
- (Qian et al. 2024) investigated how the actin cytoskeleton influences immune responses mediated by the receptor-like kinase PEPR1 following recognition of the damage-associated peptide Pep1.

## Data Availability

No new data are included in this article.
